# Formation of Size and Density Controlled Nanostructures by Galvanic Displacement

**DOI:** 10.3390/nano10040644

**Published:** 2020-03-30

**Authors:** Minh Tran, Sougata Roy, Steven Kmiec, Alison Whale, Steve Martin, Sriram Sundararajan, Sonal Padalkar

**Affiliations:** 1 Department of Mechanical Engineering, Iowa State University, Ames, IA 50011, USA; mhtran.sci.eng@gmail.com (M.T.); sroy@iastate.edu (S.R.); srirams@iastate.edu (S.S.); 2 Department of Materials Science and Engineering, Iowa State University, Ames, IA 50011, USA; skmiec@iastate.edu (S.K.); awhale@iastate.edu (A.W.); swmartin@iastate.edu (S.M.); 3 Microelectronics Research Center, Iowa State University, Ames, IA 50011, USA

**Keywords:** gold, nanostructures, surfactant, monodisperse, sensing

## Abstract

Gold (Au) and copper (Cu)-based nanostructures are of great interest due to their applicability in various areas including catalysis, sensing and optoelectronics. Nanostructures synthesized by the galvanic displacement method often lead to non-uniform density and poor size distribution. Here, density and size-controlled synthesis of Au and Cu-based nanostructures was made possible by galvanic displacement with limited exposure to hydrofluoric (HF) acid and the use of surfactants like L-cysteine (L-Cys) and cetyltrimethylammonium bromide (CTAB). An approach involving cyclic exposure to HF acid regulated the nanostructure density. Further, the use of surfactants generated monodisperse nanoparticles in the initial stages of the deposition with increased density. The characterization of Au and Cu-based nanostructures was performed by scanning electron microscopy, atomic force microscopy, UV-Visible spectroscopy, X-ray photoelectron spectroscopy, Raman spectroscopy and X-ray diffraction. The surface enhanced Raman spectroscopic measurements demonstrated an increase in the Raman intensity by two to three orders of magnitude for analyte molecules like Rhodamine 6G dye and paraoxon.

## 1. Introduction

Galvanic displacement is an electroless deposition technique utilized for the deposition of metals on semiconducting substrates [1,2]. This synthesis method has gained much attention in the past decade since it is a simple and inexpensive approach for preparing zero and two-dimensional metallic nanostructures. Moreover, it does not require complicated chemical apparatus, electrical source, or electrodes and can be performed under ambient conditions in a short timeframe. The synthesis is also applicable for substrates with complex geometries and topologies including patterned planar substrates and nanowires. Due to these advantages, there has been a surge in scientific reporting that explores the deposition of metallic nanostructures on semiconductor surfaces [1,2,3,4,5]. These deposited metallic nanostructures are either randomly distributed or specifically patterned on the substrate. There are also other literature reports that utilize this synthesis method to study fundamental growth aspects like growth behavior of metals on the underlying substrates and the characteristic of their interface [4,6,7,8]. The galvanic displacement technique has been utilized for the deposition of several metals and base metals including gold (Au) [6,7,9,10], silver (Ag) [6,8,11], platinum (Pt) [6,12,13], palladium (Pd) [6], nickel (Ni) [14], and copper (Cu) [5,14,15,16,17] on substrates like silicon (Si), germanium (Ge) [6,18] and III-V semiconductor substrates [1].

In this deposition method, the semiconducting substrate provides electrons via surface oxidation. These available electrons reduce the metal cations in solution to their metallic state, resulting in the deposition of metal on the substrate. The chemical equations below describe the galvanic displacement reactions on the Si substrate, where M denotes any metal with a standard redox potential greater than that of the substrate. Here, hydrofluoric acid (HF) acid maintains the supply of electrons by the dissolution of surface oxides on the substrate [1,9].

(1) $$Anodic:\;\;\mathrm{Si}\;\left(s\right)+6F^{-}\;\left(\mathrm{aq}\right)\to {\;\mathrm{SiF}}_{6}^{2-}\;\left(\mathrm{aq}\right)+4e^{-}$$

(2) $$Cathodic:\;{\;M}^{n+}\;\left(\mathrm{aq}\right)+\;{\mathrm{ne}}^{-}\;\;\to \;\;M\;\left(s\right)$$

(3) $$Overall:\;\;M^{n+}\;\left(\mathrm{aq}\right)+\;\mathrm{Si}\;\left(s\right)+\;6F^{-}\;\left(\mathrm{aq}\right)\;\;\to \;M\;\left(s\right)+\;{\mathrm{SiF}}_{6}^{2-}\;\left(\mathrm{aq}\right)$$

The objective of many studies on galvanic displacement has been to synthesize Au nanoparticles or films. These Au nanostructures serve as a good model system. Additionally, the redox potential of Au is greater than other desirable metals. Thus, an Au precursor can readily reduce to metallic Au, forming Au nanostructures [6,9]. Moreover, there are several potential applications for Au nanostructures in a variety of areas. Further, the plasmonic properties of Au nanostructures can be tuned by varying the size and shape of the nanostructures. These plasmonic properties can be exploited for surface enhanced Raman spectroscopy [19,20], solar cells [21], detection of bioanalytes [22], optoelectronics and also for photocatalysis [12,23]. Additionally, the knowledge acquired by thoroughly studying the galvanic displacement method for the deposition of Au nanostructures is valuable and can be applied to other metallic systems like Ag, Pt, Cu, etc. 

Lately, Cu nanostructures have been gaining importance since there are potential applications that can benefit from the plasmonic properties of Cu nanostructures. Further, Cu is a favorable material of choice due to its inherent properties like high electrical and thermal conductivity, high electromigration resistance and easy functionality [15,24,25]. Moreover, Cu is an inexpensive and abundantly available metal. Thus, due to these benefits, Cu nanostructures have been synthesized by various routes including the aforementioned method. The metallic nanostructures synthesized by galvanic displacement are often random in size and distribution [26,27]. Thus, the lack of proper control has been the limiting factor for this synthesis technique. 

In the present investigation, we successfully demonstrate a modification of the galvanic displacement method, in which surfactants like L-Cys and CTAB were incorporated to obtain controlled density, size and distribution of the deposited nanostructures. Additionally, the nanostructures can be easily removed from the substrate, making the substrate available for further depositions. Thus, the substrate becomes reusable and the removed nanostructures do not agglomerate due to the presence of the surfactants. Further, a morphological evolution of the nanostructures was observed as the synthesis process proceeded. We have synthesized Au and Cu-based nanostructures on the Si substrate. A nanostructure size as small as 7 nm has been deposited in both material systems. Our findings indicate that the surfactants, like L-cysteine and CTAB, play an important role in obtaining very dense, small size and monodisperse nanostructures. Scanning electron microscopy (SEM) was used to study the size, size distribution and morphological evolution, whereas atomic force microscopy (AFM) was used to study surface topography of the nanostructures. The compositional analysis was performed by X-ray diffraction (XRD), X-ray photoelectron spectroscopy (XPS) and Auger electron spectroscopy. The absorption properties were studied using UV-Visible spectroscopy. The surface enhanced Raman spectroscopic (SERS) measurements were performed on a Raman spectroscope. 

## 2. Materials and Methods 

The chemicals used for metal deposition included gold (III) chloride trihydrate (HAuCl_4_·3H_2_O, ≥99.9%) and cetyltrimethylammonium bromide (CTAB) (C_19_H_42_BrN, ≥99.9%) purchased from Sigma Aldrich (Milwaukee, WI, USA), cupric sulfate pentahydrate (CuSO_4_·5H_2_O, ≥98%) from Fisher Scientific (Hanover Park, IL, USA), and L-cysteine (C_3_H_7_NO_2_S, ≥98%) from EMD Millipore (Billerica, MA, USA). These chemicals were used as received. The HF acid (48%) was purchased from Macron Fine Chemicals (Center Valley, PA, USA). All solutions were prepared using deionized water. The HF acid was always contained in a polypropylene beaker for experimental purposes. The Si (100) substrate (4″ wafer, single-crystalline, n-type, *ρ* = 3–9 Ω·cm) was purchased from El-Cat Inc. (Ridgefield Park, NJ, USA). For SERS measurements, the analytes: Rhodamine 6G dye (R6G, 99%) and paraoxon-ethyl (C_10_H_14_NO_6_P, ≥90%) were purchased from Sigma Aldrich (Milwaukee, WI, USA).

**Figure 1:**
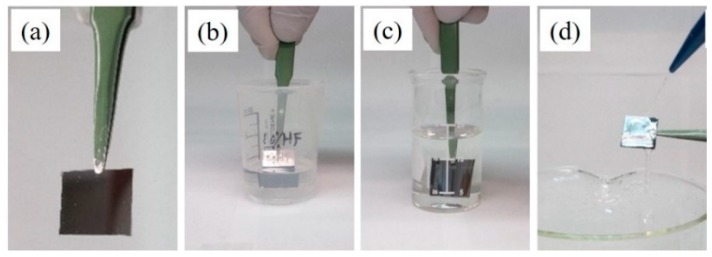
Experimental steps in the synthesis process: (**a**) cleaved and cleaned Si substrate; (**b**) immersion in 10% HF for 2 min, to dissolve the surface oxide; (**c**) immersion in 0.3 mM metal precursor solution for 5 min; (**d**) rinse sample with deionized water.

Prior to the deposition, the Si wafer was cleaved into 1.5 × 1.5 cm^2^ pieces and thoroughly cleaned using acetone followed by ethanol (100%, 200 proof) and deionized water. Each cleaning step was performed for 10 min in an ultrasonic bath in order to degrease and decontaminate the Si substrate. The clean Si substrate was left to dry on a Kimwipe. The dry Si substrate was etched in a 10% (*w/w*) aqueous HF solution for 2 min, followed by immediate immersion in an aqueous solution containing only 0.3 mM HAuCl_4_ or CuSO_4_ for 5 min, without any intermediate rinsing step. The Si substrate now had Au or Cu-based nanostructures directly deposited on it. Here, it is important to mention that the presence of residual HF on the Si substrate facilitated the direct deposition of these nanostructures. Finally, the Si substrate was thoroughly rinsed by deionized water and dried. From here on, this procedure of making nanostructures will be referred to as one deposition cycle (1X). To increase the density of the deposited nanostructures, the above procedure, (1X), was repeated up to ten times (10X). Additionally, the influence of surfactants on the nanostructure size, density and morphology was studied by incorporating the surfactants like L-cysteine (0.1 mM) and CTAB (0.3 mM) into the metal precursor solution prior to deposition. Figure 1 describes the different stages of the deposition process referred to as one deposition cycle (1X). 

The size, size distribution and morphology of the nanostructures were studied by scanning electron microscopy (SEM) using a FEI Quanta-250 SEM instrument (Thermo Fisher Scientific, Hillsboro, OR, USA) at 10 kV accelerating voltage. Topographical information of the nanostructures was obtained by atomic force microscopy (AFM) using a Veeco Dimension 3100 AFM instrument (Veeco Instruments Inc., Plainview, NY, USA), under the intermittent contact mode with a commercial Si probe (nominal radius ~8 nm, nominal frequency ~320 kHz). The scan area was 2 μm × 2 μm. To investigate the composition and crystallinity of the nanostructures, X-ray diffraction (XRD) was performed using the Siemens D500 instrument (Siemens Corporation, Washington, DC, USA). 

The absorption properties were analyzed by obtaining UV-Visible (UV-Vis) absorption spectra from a PerkinElmer Lambda 25 spectrophotometer (PerkinElmer Inc., Waltham, MA, USA). For the UV-Vis absorption measurement, the samples were prepared by sonicating the Au nanoparticles off the Si substrate in 1.5 mL of deionized water. The sonication was carried out for 3 min. The Au nanoparticles suspended in deionized water were used for UV-Vis measurements. The oxidation states of the Cu-based nanostructures were studied using X-ray photoelectron spectroscopy (XPS) by a Kratos Amicus/ESCA 3400 instrument (Kratos Analytical Inc., Chestnut Ridge, NY, USA). For the XPS data acquisition, the sample was irradiated with a 240 W Mg *K*α X-ray. The photoelectrons generated normal to the substrate were analyzed. Auger electron spectroscopy (AES) was also utilized for sample characterization.

Later in the investigation, a dye, R6G and a pesticide, paraoxon, were used to demonstrate the detection capabilities of these nanostructures via SERS measurements. The R6G dye powder (1 mg) was dissolved in 5 mL of deionized water to obtain a stock solution of 0.4 mM. Similarly, 137.6 mM paraoxon stock solution was prepared by mixing 30 μL of oily concentrated paraoxon with 0.98 mL deionized water. The samples for SERS measurements were prepared by diluting the stock solution to a desired concentration followed by drop casting 300 µL on to the nanostructures fabricated on the Si substrate. Prior to drop casting, the samples were plasma cleaned for 1 min under medium power level (11 W) by a plasma cleaner (PDC-001, Harrick Plasma, Ithaca, NY, USA). The Raman measurements were performed at room temperature on a Renishaw Dispersive Raman Spectrometer (Renishaw plc, Wotton-under-Edge, UK) with an Ar ion laser of 488 nm. An objective lens with a magnification of 50×, incident power density of 8.2 mW/cm^2^ and a total accumulation time of 2 min were used to acquire the Raman data. The Raman spectra were collected from several random locations on each sample to confirm reproducibility.

## 3. Results and Discussion

**Figure 2:**
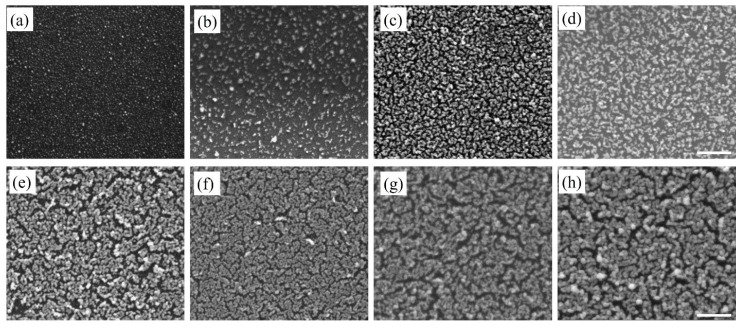
SEM images of Au nanostructures deposited on Si substrate after (**a**) first, (**b**) second, (**c**) third, (**d**) fourth, (**e**) fifth, (**f**) sixth, (**g**) eighth, and (**h**) tenth deposition cycle. The scale bar is 500 nm.

The SEM images in Figure 2 represent Au nanostructures synthesized during successive deposition cycles from 1X to 10X and show the corresponding morphological evolution of the Au nanoparticles. After the completion of 1X, the Au nanostructures appeared to be quasi-spherical in shape with an average diameter of 14 nm (Figure 2a). The nanoparticles were not deposited uniformly over the entire Si substrate. With the completion of 2X, the Au nanostructures were a combination of quasi-spherical nanoparticles and elongated branched nanostructures. The additional deposition cycles produced more nanostructures on the substrate and the morphological evolution of the deposited Au nanostructures was evident. Up to 5X, Au nanostructures appeared as chains of nanoparticles forming a continuous network, and the average width of the Au nanostructures increased from 18 nm to 32 nm. After the completion of 6X, the chains of nanoparticles coalesced forming a coating on the Si substrate, with small uncoated regions. With the increase in the deposition cycles, the nanostructures increased in size due to agglomeration and the narrow uncoated substrate regions remained primarily unchanged. The uncoated regions are due to multiple factors such as lack of accessibility for the HF acid during the synthesis process and coalescence of Au nanostructures because of the surface energy minimization [28]. 

**Figure 3:**
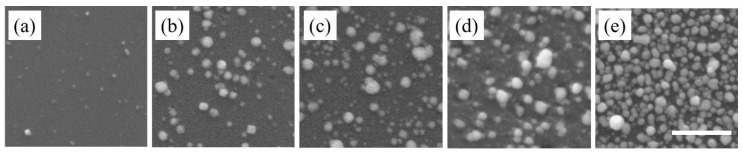
SEM images of Cu-based nanostructures deposited on Si substrate after (**a**) first, (**b**) second, (**c**) third, (**d**) fourth and (**e**) fifth deposition cycle. The scale bar is 500 nm.

Along with the deposition of Au nanostructures, Cu-based nanostructures were also deposited using the galvanic displacement technique (Figure 3). Unlike Au nanostructures, the deposited Cu-based nanostructures were sparse during the initial deposition cycles. This sparse deposition was due to the lower redox potential of Cu compared to Au. With increases in the deposition cycles, the number density of Cu-based nanostructures increased. Moreover, the morphological evolution did not follow a similar trend to that established for the Au nanostructures. There was no evidence of nanoparticle chain formation since each nanoparticle appeared as an individual entity. The average size of the Cu-based nanostructures increased from 23 to 42 nm with increasing deposition cycles. The difference in the morphological evolution between Au and Cu-based nanostructures was primarily due to the difference in their surface energies. The Au nanostructures appeared like a network of nanoparticle chains, while the Cu-based nanostructures appeared more spherical with limited agglomeration. Reports in the literature have demonstrated that the surface energies of low index planes for Au are much lower than that of Cu. Thus, surface energies of low index planes played a critical role in the resulting morphologies [6,9,29].

From the SEM images, it was clear that the deposition of Au and Cu-based nanostructures on the Si substrate occurred via the Volmer–Weber growth mode. The number density of the deposited nanostructures in the first cycle appeared to be higher than the immediate successive cycle. This initial decrease in the number density was attributed to two factors: weak adhesion between the deposited nanostructures and the underlying Si substrate and the lack of formation of the critical nanoparticle size [9]. Here, it is important to note that in the Volmer–Weber growth mode, the binding energy between atoms within the deposited nanostructures is higher than that with the substrate. However, with increase in the deposition cycles the number density increased, which occurred after the stabilization of the initial critical nanoparticle size. Since this deposition process occurred via nucleation and growth, at every deposition cycle there were additional nucleation events and growth of existing nanostructures. This led to the formation of polydisperse nanostructures. With further deposition, the availability of the Si surface decreased. This was an important aspect, since the formation of nanostructures was only possible if the Si surface was accessible for reaction with HF acid. Thus, the two limiting factors of this deposition process were the formation of polydisperse nanostructures and the lack of accessibility to narrow Si surfaces, between nanostructures, after several deposition cycles affecting complete substrate coverage. The first limiting factor of polydispersity was addressed in this investigation. 

**Figure 4:**
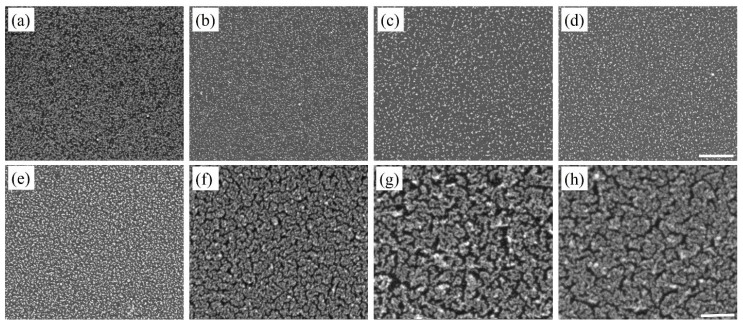
SEM images of Au nanostructures deposited on the Si substrate after (**a**) first, (**b**) second, (**c**) third, (**d**) fourth, (**e**) fifth, (**f**) sixth, (**g**) eighth, and (**h**) tenth deposition cycle with L-Cys added as surfactant. The scale bar is 500 nm.

**Figure 5:**
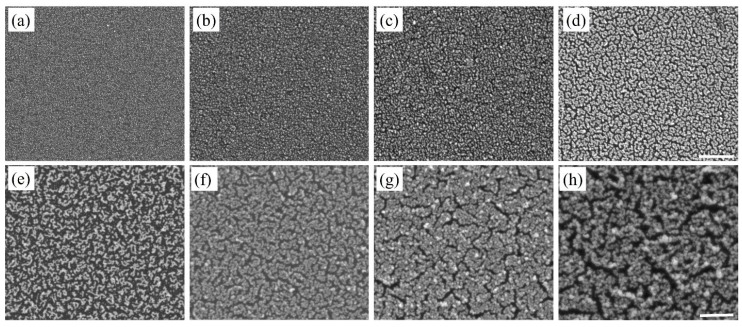
SEM images of Au nanostructures deposited on Si substrate after (**a**) first, (**b**) second, (**c**) third, (**d**) fourth, (**e**) fifth, (**f**) sixth, (**g**) eighth, and (**h**) tenth deposition cycle with CTAB added as surfactant. The scale bar is 500 nm.

**Figure 6:**
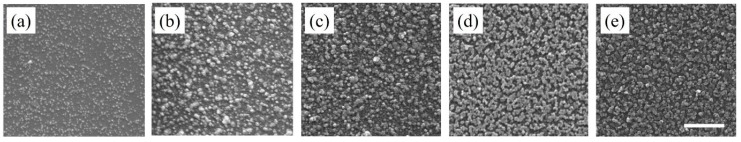
SEM images of Cu-based nanostructures deposited on Si substrate after (**a**) first, (**b**) second, (**c**) third, (**d**) fourth, (**e**) fifth deposition cycle with L-Cys added as surfactant. The scale bar is 500 nm.

**Figure 7:**
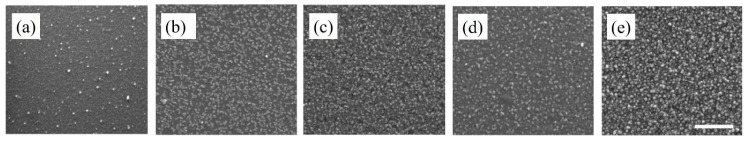
SEM images of Cu-based nanostructures deposited on Si substrate after (**a**) first, (**b**) second, (**c**) third, (**d**) fourth, (**e**) fifth deposition cycle with CTAB added as surfactant. The scale bar is 500 nm.

In solution-based synthesis of nanostructures, the use of surfactants is commonplace, which addresses issues of polydispersity. Surfactants like L-cysteine (L-Cys) and cetyl trimethylammonium bromide (CTAB) have been used to stabilize nanoparticles in solutions [30,31] and on templates [32,33]. The ability of surfactants to selectively adsorb on certain crystal facets facilitates the control of size and morphology of the synthesized nanostructures. Thus, the commonly used surfactants, L-Cys and CTAB, were used with the objective to overcome the problem of polydispersity due to this deposition process. The surfactants were added independently into the Au and Cu precursor solutions and the final solution was used for the deposition of Au and Cu-based nanostructures. Figure 4, Figure 5, Figure 6 and Figure 7 show SEM images of Au and Cu-based nanostructures deposited in the presence of L-Cys and CTAB. Furthermore, Figure S1 shows our results of additional experiments performed where the sequence of adding the surfactants to the deposition process was altered to investigate their effects on Si substrate and the metallic nanostructures. The strong influence of the surfactants was demonstrated by decreased size and increased density of nanostructures during the initial deposition cycles (compared with Figure 2 and Figure 3). Literature reports state that L-Cys promotes growth of Au nanostructures in <111> direction by adsorbing on high-index planes [34,35]. It is also known that CTAB has a tendency to adsorb on the (100) and (110) planes of fcc crystal structure [36,37]. The overall reduction in size indicated that surfactants adsorbed on the nanostructures and slowed down the addition of new metallic atoms.

Additionally, the two surfactants, L-Cys and CTAB, are reported to act as mild reductants [31,34,38,39,40]. The thiol group of L-Cys is capable of donating one electron to reduce metal ions. The metal ions bind to the remaining L-Cys to form a complex. Thus, several of the metal ion—cysteine complexes can undergo polymerization forming macromolecular structures, which can be degraded by the electrons from the Si substrate. Consequently, the metal ions reduce to metallic Au (0) or Cu (0). Thus, the additional reduction function of L-Cys surfactant and the ability to concentrate metal (I) ions within the macromolecular structure facilitated the galvanic displacement process to form highly dense nanostructures with reduced size (Figure 4 and Figure 6) [41]. 

Similarly, CTAB behaves as a mild reductant. The negatively charged Au precursor (AuCl_4_^−^) binds to the cationic CTAB surfactant via electrostatic interactions. The degradation of the long carbon chains in CTAB facilitates the reduction of the metal ions [42,43]. For Cu-based nanostructures, the Cu^2+^ cation may not electrostatically interact with CTAB head group. However, the Cu^2+^ ions could bind with CTAB via intermediate Br^−^ counterions and thus participate in the reduction process. Thus, the reduction in nanostructure size in the presence of surfactants conformed to the literature reports and demonstrated a promising approach to overcome polydispersity in the galvanic displacement method, where nanostructures were directly deposited on the Si substrates (Figure 4, Figure 5, Figure 6 and Figure 7). However, detailed studies are warranted to elucidate the reduction mechanism of metal ions to metallic Au (0) or Cu (0) in the presence of surfactants like L-Cys and CTAB.

Further, after the first deposition cycle, the nanostructure number density exhibited a large increase compared to the non-surfactant counterparts. It was observed that the number density of Cu-based nanostructures was smaller than Au nanostructures (Figure S2). This smaller number density was attributed to two factors: the lower redox potential of Cu compared to Au [6,9] and high surface energy of low index Cu planes compared to Au [29]. Additionally, the nanostructures underwent morphological evolution, with an increase in deposition cycles, exhibiting similar trends to their non-surfactant counterparts (Figure 4, Figure 5, Figure 6 and Figure 7). Table 1 summarizes the average size of Au and Cu-based nanostructures in the presence and absence of surfactants, based on the analysis of at least 500 nanoparticles from multiple SEM images. It is clear that Au nanostructures were consistently smaller than the Cu-based nanostructures. It also confirmed the strong influence of surfactants on the nanostructure size.

**Figure 8:**
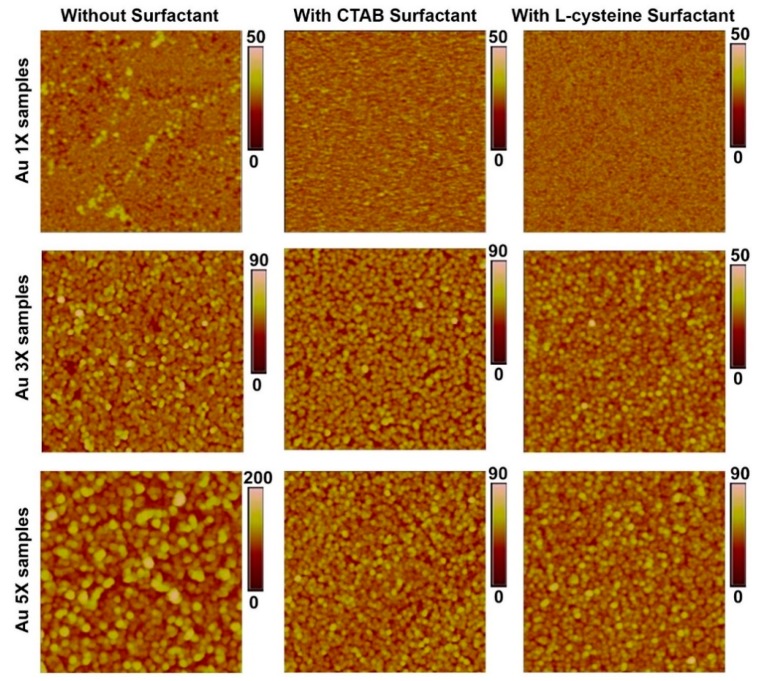
Representative AFM topography maps of the Au samples after one (1X), three (3X), and five (5X) deposition cycles. Scan area of 2 μm × 2 μm. Height scale is in nm.

**Figure 9:**
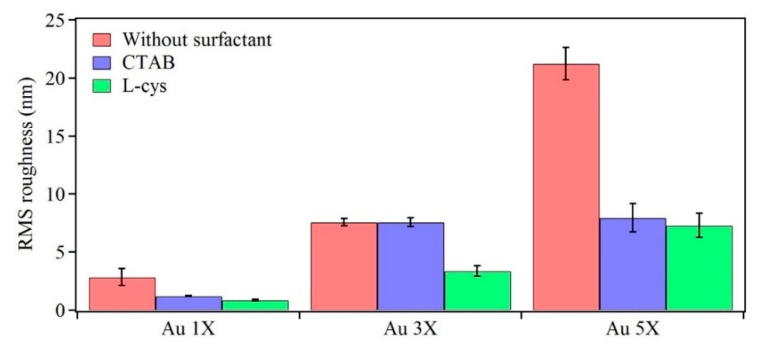
RMS roughness of Au samples after one (1X), three (3X), and five (5X) deposition cycles. Mean values from five different locations on each sample are shown. Error bars represent 95% confidence intervals.

The topological information of these nanostructures was obtained through AFM studies. Figure 8 shows topographical images of Au samples after one, three, and five deposition cycles. It can be observed that increasing the number of deposition cycles resulted in Au nanostructures with increasing height and lateral size, thus, supporting the SEM data. When surfactants were added, they alleviated aggregation and agglomeration, as indicated by smoother surfaces. Figure 9 shows the root mean square (RMS) roughness values for the prepared samples. It can be observed that increasing the number of deposition cycles (from 1X to 5X) increased the surface roughness of the samples due to the increasing size of the Au nanostructures. The data confirms the visual observations from the SEM images regarding the role of surfactants in reducing aggregation and agglomeration, thereby leading to a more homogeneous surface due to L-Cysteine and CTAB.

**Figure 10:**
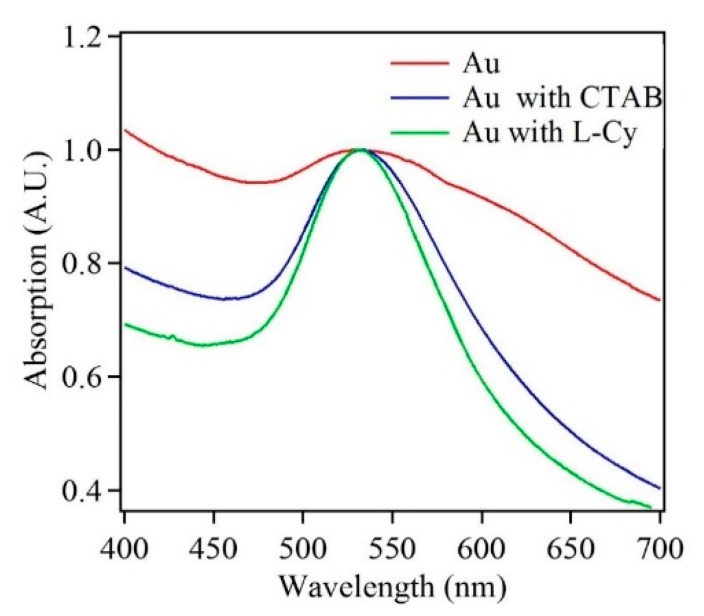
Absorption spectra of Au nanoparticles after the first deposition cycle in the absence and presence of surfactants.

Along with the above measurements, the absorption properties of Au nanostructures were analyzed for the first deposition cycle. Figure 10 shows the UV-Vis spectra for Au nanoparticles deposited in the absence and presence of either surfactant (L-Cys or CTAB). The surface plasmon resonance (SPR) for gold appeared between 500–600 nm and was attributed to the transverse plasmon mode [44]. The UV-Vis spectra for Au nanoparticles in the absence of surfactants exhibited a broad spectrum with two maxima centered at ~532 nm and ~610 nm, which confirmed the formation of Au and presence of at least two nanostructure sizes. The UV-Vis spectra for Au samples in the presence of surfactants like L-Cys and CTAB indicated a narrower line width with a maximum at 532 nm. The comparison of UV-Vis spectra indicated that Au nanostructures deposited in the absence of surfactants were polydisperse, whereas the ones deposited in the presence of surfactants were monodisperse. The SEM images confirm the UV Vis data for the Au nanostructures (Figure 2a, Figure 4a and Figure 5a).

The absorption data was also obtained for Au nanostructures samples from all deposition cycles in the presence and absence of surfactants. With the increase in deposition cycles, the absorption maximum underwent a red shift along with line broadening. These absorption results conformed to the reports in literature. An increase in Au nanostructure size was due to increased aggregation, and decrease in nanostructure distances, which led to increased interparticle plasmon coupling. In addition, the overall trend of increasing line broadening indicated nanostructure dispersity with the number of deposition cycles. Figure S3 and Table S1 show additional absorption data for all deposition cycles.

**Figure 11:**
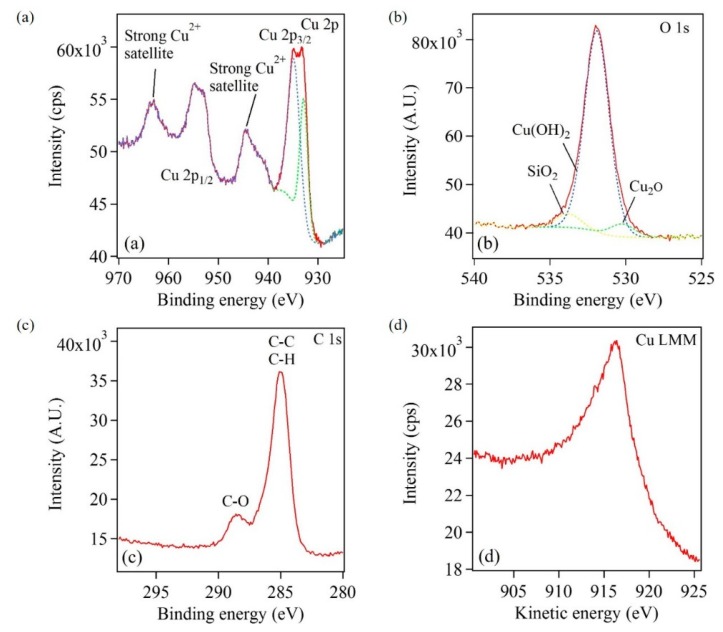
XPS spectra showing (**a**) Cu 2p peaks corresponding to different oxidation states of Cu, (**b**) O 1s peaks corresponding to different oxide species, and (**c**) C 1s peaks corresponding to various carbon bonds (**d**) AES spectrum showing Cu LMM peak that resemble Cu(OH)_2_ and Cu_2_O. The dashed curves in (**a**) and (**b**) represent Gaussian curve fits.

The Cu-based nanostructures were characterized by XPS to obtain information of the surface composition. The Casa XPS processing software was used to analyze the XPS data. Figure 11 shows XPS spectra and an AES spectrum of Cu-based nanostructures. The broad peak of Cu 2p_3/2_ in Figure 11a was composed of two maxima at 932.9 and 934.9 eV. Two Gaussian curves were fit to this broad peak and are represented by dashed lined peaks. Similarly, a broad peak of Cu 2p_1/2_ was composed of two maxima at 952.2 and 954.7 eV. Additionally, two strong Cu^2+^ satellite peaks were observed in the XPS spectrum. The presence of Cu 2p_3/2_ (934.9 eV), Cu 2p_1/2_ (954.7 eV) and satellite peaks indicate the presence of Cu(OH)_2_ on the surface of the Cu-based sample [45,46]. Furthermore, while Cu 2p_1/2_ and Cu 2p_3/2_ peaks at 952.2 and 932.9 eV, respectively, can confirm the presence of metallic Cu, they may imply the presence of Cu_2_O as well, since Cu 2p peaks of Cu and Cu_2_O have the same binding energies [45,46]. An AES spectrum (Figure 11d) was obtained for the Cu-based sample to further elucidate the chemical composition of Cu-based sample [46,47,48]. A broad Cu LMM peak centered at 916.4 eV was observed in the AES spectrum. This broad peak was attributed to an overlap of two maxima at 916.3 and 916.7 eV, which correspond to the presence of Cu(OH)_2_ and Cu_2_O respectively [47,48].

Further, the location of the O 1s peak was analyzed to study the nature of the oxide species. Figure 11b showed a broad peak that was composed of three maxima at 530.3, 531.9, and 533.8 eV, which represent characteristic oxides of Cu_2_O, Cu(OH)_2_, and SiO_2_, respectively [49]. Here, the XPS data was fit by three Gaussian peaks. This is represented by the dotted lined peaks in Figure 11b. Figure 11c provided information on surface adsorbed species. The strong peak at 285.1 eV matched with C–C and C–H bonds and peak at 288.6 eV was assigned to C–O bond, which arise from surface contaminants [49,50]. Thus, from the XRD (Figure S4), XPS and AES characterization of Cu-based nanostructures, it was inferred that the Cu-based nanostructures were composed of a metallic Cu core with a Cu_2_O and Cu(OH)_2_ outer layer. Similar systems of copper core and very thin copper oxide shell have been described in the literature [51,52]. 

**Figure 12:**
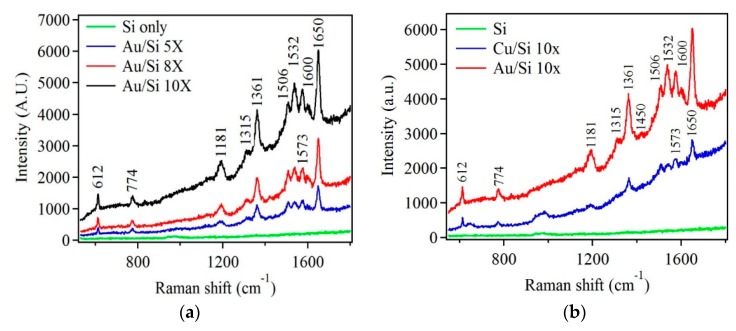
(**a**) Raman spectra of R6G on Si, and Au nanostructures samples after five, eight, and ten deposition cycles. (**b**) Raman spectra showing R6G modes for Au and Cu-based nanostructures. R6G concentration was 10^−5^ M.

Finally, scattering properties were evaluated for Au and Cu nanostructures via Raman spectroscopic measurements to test their capabilities in the field of Surface Enhanced Raman Spectroscopy (SERS). Literature reports have established Au as an ideal candidate for SERS due to its stability, biocompatibility, and easy surface treatment [53] While charge transfer between adsorbed molecules and Au contribute to signal enhancement, the effect of localized surface plasmon resonance due to Au nanostructures was an exclusively dominating mechanism [54,55]. Figure 12a shows Raman spectra of R6G dye that was drop cast on Au nanostructure samples having five, eight and ten deposition cycles. For comparison, a reference Si substrate with the same amount of R6G was prepared. The reference sample did not detect any Raman modes. The Au nanostructure samples exhibited several strong Raman modes indicating the presence of R6G dye and represented several important vibrational modes of R6G. It was observed that with increase in deposition cycles the Raman signal intensity increased. Similar experimental conditions were employed to obtain Cu-based nanostructure samples for the detection of R6G. Figure 12b shows Raman spectra of R6G dye obtained from the Cu-based nanostructure sample. It was observed that the Raman intensity was weaker than that of Au samples. However, the important Raman modes of R6G were observed for the Cu-based samples.

**Figure 13:**
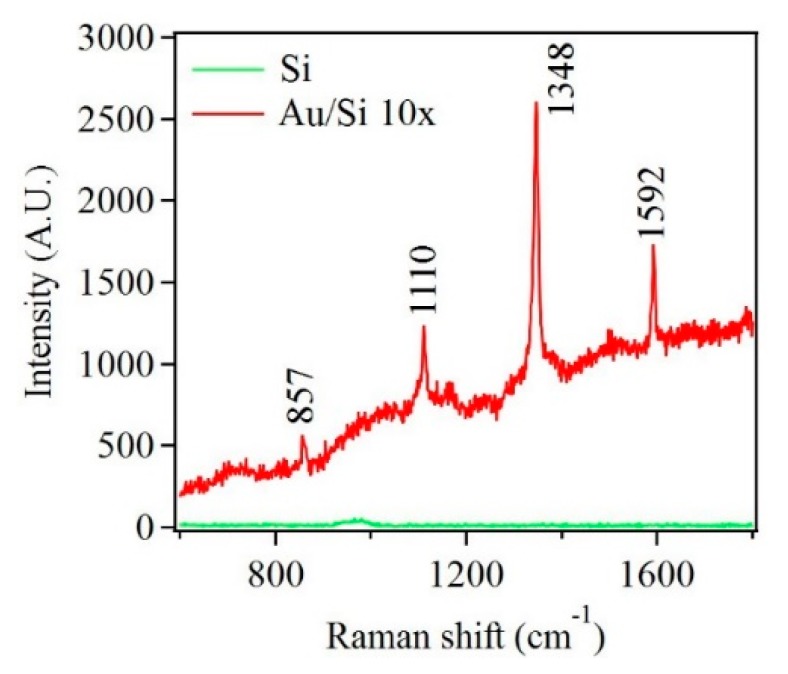
Raman spectra of paraoxon adsorbed on Si substrate and Au nanostructures deposited on Si after ten deposition cycles.

The second SERS measurements were performed on similar samples as mention above but in the presence of a pesticide molecule, paraoxon, as the analyte. The objective of this part of the investigation was to test whether the fabricated samples could detect the presence of a smaller molecule. Paraoxon was chosen since it is a hazardous chemical used in various forms as a pesticide in agriculture and is smaller in comparison to the model R6G dye molecule [56]. Figure 13 shows Raman spectra obtained from the Au nanostructure sample in the presence of paraoxon. The spectrum exhibited four strong Raman modes indicating the presence of paraoxon [57,58]. Although the presence of paraoxon was successfully detected, the concentration of paraoxon was relatively high (10^−2^ M). It is also important to note that the wavelength used for SERS measurements (488 nm) was not ideal for Au and Cu-based nanostructures. With a correct choice of wavelength, the samples are expected to demonstrate better SERS performance. However, this experimental setup for SERS was used as a proof of concept for these nanostructures. Thus, the nanostructures fabricated by this modified galvanic displacement technique show promise and present scope for further optimization. 

## 4. Conclusions

In summary, a modified galvanic displacement method was presented. Here, Au and Cu-based nanostructures were deposited on Si (100) substrate using limited and cyclic HF exposure. The density, size, size distribution and morphology of the nanostructures were controlled by varying the deposition cycles and incorporating surfactants like L-Cys and CTAB in the deposition process. The presence of surfactants produced monodisperse, highly dense, extremely small nanostructures during the initial deposition cycles (1X–5X). Morphological evolution, during the deposition cycles, led to the formation of continuous network of nanostructures. These nanostructure networks were capable of detecting organic molecules of varying sizes. The initial monodisperse nanostructures can find potential use in catalysis or as seed nanoparticles to initiate growth on organic substrates. The continuous network nanostructures can be used in sensing as mentioned above and in applications for entrapment of molecules for cleaning purposes or drug release. Thus, these nanostructures are of value at every stage of the deposition cycle. 

## Figures and Tables

**Table 1:** Average nanostructure size of Au and Cu-based samples.

Sample	Nanostructure Size after One Deposition Cycle (nm)		
	Absence of Surfactant	Presence of L-Cys	Presence of CTAB
Au	11 ± 4	8 ± 3	9 ± 3
Cu-based	23 ± 5	18 ± 7	19 ± 5

## Supplementary Materials

The following are available online at https://www.mdpi.com/2079-4991/10/4/644/s1. Figure S1: SEM images of Au nanostructures deposited on Si substrate after one deposition cycle, after sequential exposure to (a) L-Cys then HAuCl_4_, (b) L-Cys then mixture of HAuCl_4_ and L-Cys, (c) CTAB then HAuCl_4_, and (d) CTAB then mixture of HAuCl_4_ and CTAB. The scale bar is 500 nm, Figure S2: The number densities of Au and Cu-based nanoparticles with and without surfactant after one deposition cycle, Figure S3: UV-Vis spectra of Au samples (a) without surfactants, (b) with L-Cys and (c) with CTAB. The first deposition cycle is 1X and the fifth deposition cycle is 5X, Figure S4: XRD plots of (a) Au and (b) Cu-based nanostructures on Si substrates. The plots suggest FCC structures for Au and Cu-based nanostructures, Table S1: SPR peak positions of Au samples in the absence and presence of surfactants, recorded corresponding to each deposition cycle.
