# Supplementary material for: Formation of Size and Density Controlled Nanostructures by Galvanic Displacement
Source: Nanomaterials (Basel). 2020 Mar 30;10(4):644. doi: 10.3390/nano10040644 (PMC7221692; doi:10.3390/nano10040644)
Supplement: Supplementary file 1 [file nanomaterials-10-00644-s001.pdf]

nanomaterials

Formation of Size and Density Controlled Nanostructures by Galvanic Displacement – Supplementary Information

Minh Tran [1], Sougata Roy [1], Steven Kmiec [2], Alison Whale [2], Steve Martin [2], Sriram Sundararajan [1] and Sonal Padalkar [1,3,*]

[1] Department of Mechanical Engineering, Iowa State University, Ames, IA 50011, USA; mhtran.sci.eng@gmail.com (M.T.); sroy@iastate.edu (S.R.); srirams@iastate.edu (S.S.)
[2] Department of Materials Science and Engineering, Iowa State University, Ames, IA 50011, USA; skmiec@iastate.edu (S.K.); awhale@iastate.edu (A.W.); swmartin@iastate.edu (S.M.)
[3] Microelectronics Research Center, Iowa State University, Ames, IA 50011, USA
* Correspondence: padalkar@iastate.edu; Tel.: +1-515-294-6066

To investigate whether surfactant adsorption to Si substrate would affect the overall density and size distribution of the nanostructures, extensive experiments were performed. Figure S1 shows SEM images of Au samples and the incorporation of surfactants at various stages of the deposition process.

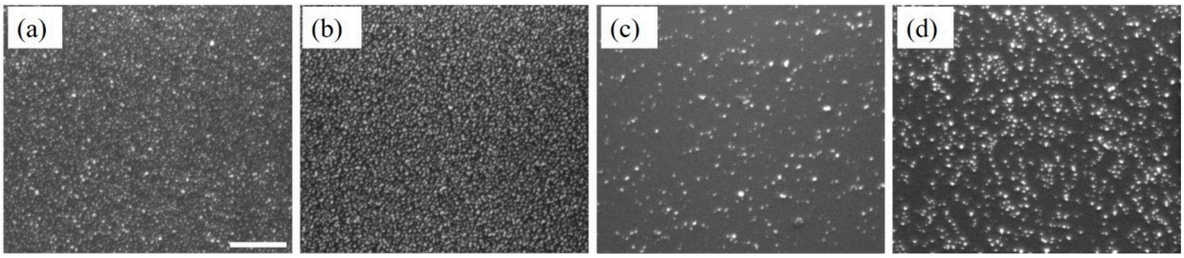

Figure S1. SEM images of Au nanostructures deposited on Si substrate after one deposition cycle, after sequential exposure to (**a**) L-Cys then $HAuCl_4$, (**b**) L-Cys then mixture of $HAuCl_4$ and L-Cys, (**c**) CTAB then $HAuCl_4$, and (**d**) CTAB then mixture of $HAuCl_4$ and CTAB. The scale bar is 500 nm.

We conducted three sets of experiments for one deposition cycle. In the first set of experiment, Si substrate was immersed in 10% HF solution for 2 min., followed by immersion in 0.1 mM L-Cys solution for 5 min. and then treating the substrate with 0.3 mM $HAuCl_4$ solution for 5 min. In the second set of experiment, the experimental steps remained the same, except that the final solution contained a mixture of 0.3 mM $HAuCl_4$ and 0.1 mM L-Cys. In the third set of experiments, surfactant CTAB was used in place of L-Cys while other conditions and reagents were unchanged.

When L-Cys was used separately prior to $HAuCl_4$ solution (Figure S1a), the resulting Au nanostructures appeared to be similar in size and density to those obtained without the surfactant L-Cys (Figure 2a). When L-Cys was incorporated in two separate steps during deposition, a large increase in the density of the nanostructures was observed (Figure S1b). Similar trends were observed when surfactant CTAB was used in the deposition process. It is also important to note that there were many Au nanoparticles in the CTAB samples, which were not clearly distinguishable from the SEM images (Figures S1c,d). Thus, it can be concluded that adsorption of either L-Cys or CTAB to Si substrate was negligible and, therefore, did not compete with Au atoms for nucleation sites. Furthermore, the results shown in Figure S1, confirmed the importance of including L-Cys or CTAB in the metal precursor solution in controlling the density of the deposited nanostructures. Thus, similar procedure was adopted in the experiments described in this investigation.

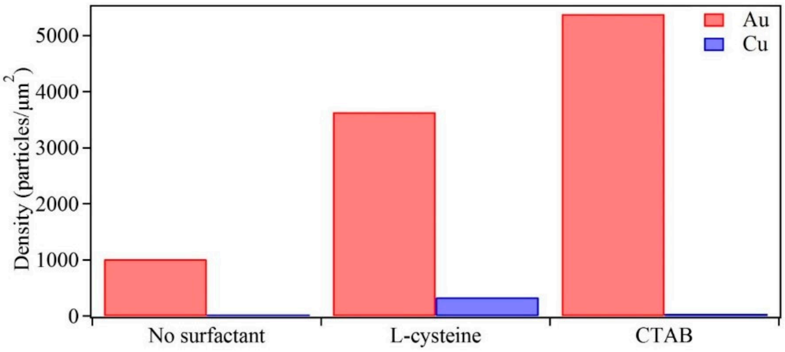

Figure S2. The number densities of Au and Cu-based nanoparticles with and without surfactant after one deposition cycle.

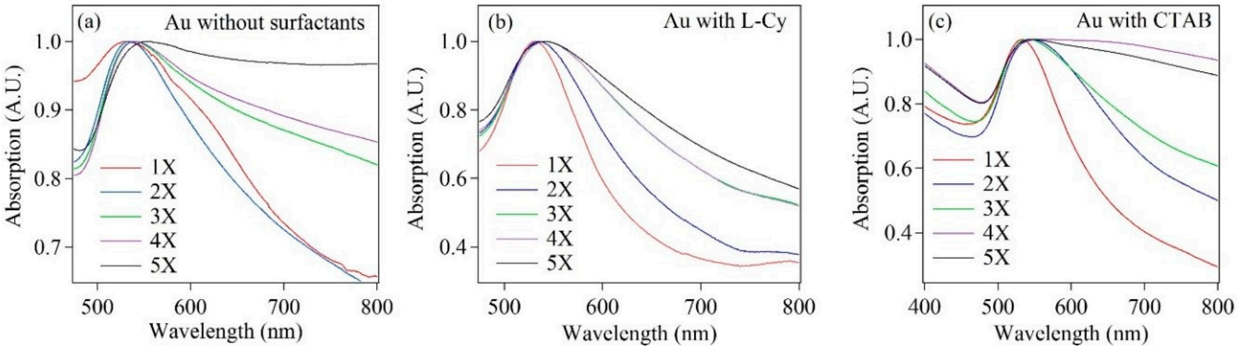

Figure S3. UV-Vis spectra of Au samples (**a**) without surfactants, (**b**) with L-Cys and (**c**) with CTAB. The first deposition cycle is 1X and the fifth deposition cycle is 5X.

Table S1. SPR peak positions of Au samples in the absence and presence of surfactants, recorded corresponding to each deposition cycle.

**No. of deposition cycles**	**SPR peak position for Au nanostructures (nm)**		
	**Absence of surfactant**	**Presence of L-Cys**	**Presence of CTAB**
1X	534	532	534
2X	534	535	546
3X	539	544	543
4X	541	544	546
5X	554	542	546

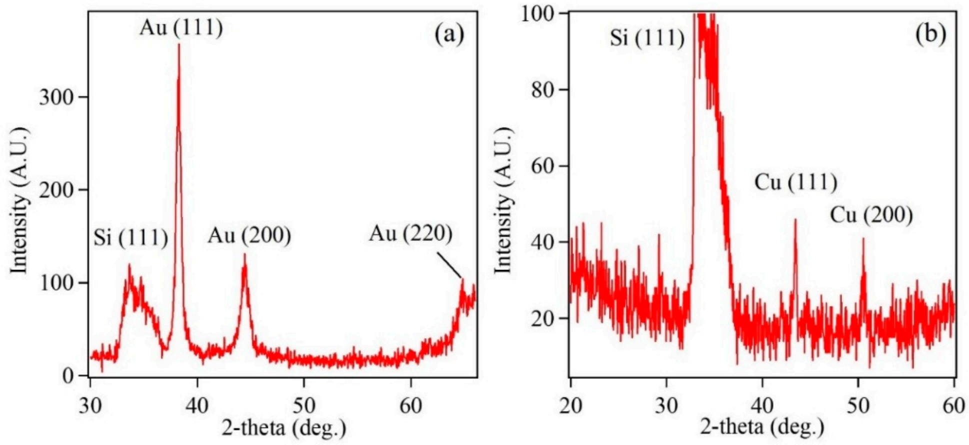

Figure S4. XRD plots of (**a**) Au and (**b**) Cu-based nanostructures on Si substrates. The plots suggest FCC structures for Au and Cu-based nanostructures.

The XRD plots of Au and Cu-based nanostructures deposited on Si substrates after five deposition cycles are presented in Figures S4a and S4b, respectively. The XRD peaks were indexed and matched well with Au nanostructures indicating a face-centered cubic (fcc) structure of Au [1].The peak with the highest intensity was centered at $2\theta = 38.3°$ and was indexed as Au (111) crystal plane, indicating a preferred orientation of the deposited Au nanostructures. For the Cu-based nanostructures, we observed two weak peaks located at $2\theta = 43.45°$ and 50.55°, which corresponded to Cu(111) and Cu(200) crystal planes of fcc Cu, respectively [2–4]. Thus, based on the XRD data, the composition of Cu-based nanostructures consists of fcc Cu. It was noted that, both XRD plots, exhibited Si(100) peak at around 70° (not shown) and Si (111) at around 35°, arising from the underlying substrate [5].

References

1. Nalawade, P.; Mukherjee, T.; Kapoor, S. Green Synthesis of Gold Nanoparticles Using Glycerol as a Reducing Agent. *ANP* **2013,** *2*, 78–86.
2. Wu, S.; Chen, D. Synthesis of high-concentration Cu nanoparticles in aqueous CTAB solutions. *J. Colloid Interf. Sci.* **2004,** 273, 165–169.
3. Panigrahi, S.; Kundu, S.; Basu, S.; Praharaj, S.; Jana, S.; Pande, S.; Ghosh, S.; Pal, A.; Pal, T. Cysteine functionalized copper organosol: synthesis, characterization and catalytic application. *Nanotechnology* **2006,** *17*, 5461–5468.
4. Bicer, M.; Sisman, I. Controlled synthesis of copper nano/microstructures using ascorbic acid in aqueous CTAB solution. *Powder Technol.* **2010,** *198*, 279–284.
5. Lam, Y.; Zheng, H.; Tjeung, R.; Chen, X. Seeing the invisible laser markings. *J. Phys. D Appl. Phys.* **2009,** *42*, 042004.

© 2020 by the authors. Submitted for possible open access publication under the terms and conditions of the Creative Commons Attribution (CC BY) license (http://creativecommons.org/licenses/by/4.0/).
